# A Semicircular Incision in the Superior Umbilical Fold for SILS Preserves the Umbilical Profile

**DOI:** 10.1155/2012/412623

**Published:** 2012-12-12

**Authors:** S. C. Blackburn, S. D. Adams, A. A. Mahomed

**Affiliations:** Department of Paediatric Surgery, Royal Alexandra Children's Hospital, Eastern Road, Brighton BN2 5BE, UK

## Abstract

*Background*. Single Incision Laparoscopic Surgery (SILS) has been highlighted in the recent literature as a means of performing a range of common, minimal access, paediatric surgical procedures. The primary attraction is the absence of visible scarring. *Aim*. This study aims to describe a cosmetically advantageous means of SILS port placement in children, which preserves the umbilical profile. *Methods*. We describe a paediatric case series utilising a semicircular incision in the superior umbilical fold for SILS procedures. The linea alba is exposed over 2 cm just superior to the umbilical ring and stay sutures are applied. A vertical incision is made over this distance without entering the umbilical ring. Data were recorded prospectively in a Microsoft Excel database. *Results*. Twenty-one cases were performed in a 1-year period. Ten appendicectomies, 5 ovarian/paraovarian cystectomies, 2 Palomo procedures, 3 nephrectomy/heminephrectomies, and 1 Meckel's diverticulectomy were performed. There was 1 wound infection. No incisional hernias occurred. *Discussion*. We believe that our technique, which maintains the integrity of the umbilical ring and allows preservation of the umbilical profile, offers a distinct cosmetic advantage over other incisions for SILS which distort it. *Conclusion*. We have demonstrated the aesthetic benefits of utilising a superior umbilical-fold incision for SILS in children.

## 1. Introduction

Single incision laparoscopic surgery (SILS) has become established in recent paediatric surgical practice. SILS has been used in children to perform: splenectomy, appendicectomy, inguinal hernia repair, pyloromyotomy, cholecystectomy, and fundoplication [1, 2].

In the hands of experienced minimal access surgeons, SILS has the advantage of limiting the number of visible incisions, potentially decreasing trauma to the abdominal wall, which has the potential to lead to shortened hospital stay and faster recovery [3]. Another potential advantage of SILS is that it utilises a skill set which surgeons performing paediatric laparoscopy already possess. This is in contrast to natural orifice transluminal endoscopic surgery (NOTES), which requires an entirely different skill set.

A variety of techniques have been described to access the abdomen in children. The same proprietary devices as are used in adult practice can be applied to paediatric patients. The Covidien SILS port (Covidien, Dublin, Ireland), Advanced Surgical Concepts Triport (Advanced Surgical Concepts, Bray, Ireland), and the Uni-X device (Pnavel Systems, Brooklyn, NY) have all been used in children [2]. 

The majority of authors describing access for SILS ports in paediatric practice have described the use of a transumbilical incision. A transumbilical incision can be extended, without breaching the limits of the umbilicus, by extension of the skin incision in a “Yin-Yang” configuration, in which a vertical incision in the umbilicus is extended circumferentially along the margins of the umbilicus at either end [1]. Unfortunately, this approach with an incision made through the middle of the umbilical ring disrupts its configuration. In reflection on our experience in performing transumblical pyloromyotomy in infants [4], we wondered whether our previously described technique for opening the abdomen could be applied to the placement of a SILS port, offering the cosmetic advantage of preserving the integrity of the umbilical ring and preserving the umbilical profile.

## 2. Methods

A prospective record of a single surgeon's experience of this technique was kept over a one-year period. The superior umbilical fold incision was employed for abdominal access in all patients undergoing SILS procedures. The same technique for placement of the SILS port was employed in all patients.

Data were collected prospectively in a Microsoft Excel database. Patients were followed up in outpatients 6–12 weeks following surgery and the wound reviewed. Patients were also questioned about the occurrence of wound complications.

### 2.1. Surgical Technique

The patient is placed supine, and an intravenous dose of flucloxacillin or coamoxiclav is given if antibiotic therapy has not already been instituted. A hemicircumferential incision is placed in the superior umbilical fold. The linea alba is exposed cranially and is opened in the midline between stay sutures. The inferior extent of the incision in the linea alba is often taken to the left of the umbilical ring leaving a 2 to 3 mm margin of sheath on the latter in order to facilitate effective closure. The peritoneum is entered, and once the surgeon's index finger is able to pass comfortably into the opening, a SILS port (Covidien, Dublin, Ireland) or Triport (Advanced Surgical Concepts, Bray, Ireland) is introduced into the abdomen with the aid of a Robert's or similar clamp. Once the SILS procedure is complete, the incision is closed with a single, continuous, PDS suture appropriate to the size of the child. The skin is closed with an interrupted subcuticular 5–0 vicryl rapide suture (Ethicon, Edinburgh, Scotland).

## 3. Results

Twenty-one cases underwent a SILS procedure during the study period. All cases were completed successfully using a SILS technique (Table 1). A SILS port (Covidien, Dublin, Ireland) was used in 19 cases and a Triport (Advanced Surgical Concepts, Bray, Ireland) in 2 cases. The mean age of the patients was 14 years (range 7–19 years), and 14 of the patients were males.

**Figure 1:**
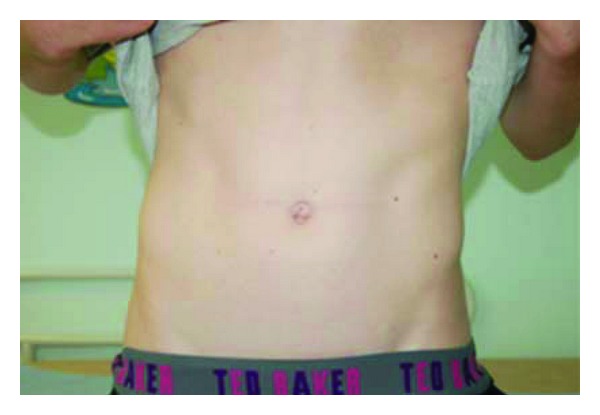
Clinical photograph of a 14-year-old patient 12 weeks following a SILS Palomo procedure.

**Figure 2:**
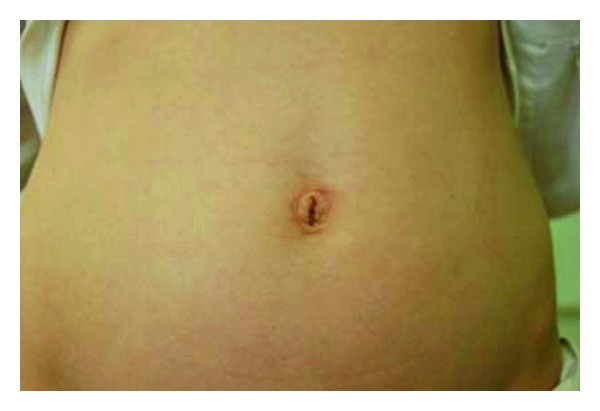
Clinical photograph of a 17-year-old patient 3 months following a SILS appendicectomy. Residual oedema of the superior aspect of the umbilicus is still evident.

One wound infection occurred in a teenager with perforated appendicitis. This was successfully managed conservatively, using dressings and intravenous antibiotics. No incisional hernias were observed at followup. Cosmetic results were favourable in all patients at followup. Representative clinical photographs are shown to illustrate this (Figures 1 and 2).

## 4. Discussion

We describe a series of patients undergoing SILS procedures in whom an incision in the superior umbilical fold was employed. This technique was successful in allowing access for the SILS port and producing good cosmetic results, with only one wound-related complication in a patient with perforated appendicitis.

The previously described “Yin-Yang” incision has the disadvantage, we believe, of disturbing the integrity of the umbilical ring and leading to loss of the umbilical profile. Having previously demonstrated that the superior umbilical fold incision could successfully be used to access the peritoneal cavity when performing a pyloromyotomy, we have now shown that this technique can be used to successfully place a SILS port, leading to a favourable cosmetic result in which the umbilical ring is preserved.

The disadvantage of this technique in smaller children, infants, and in those with a featureless umbilicus is that all proprietary devices for SILS access require a minimum incision of 20 mm to be inserted [5]. This would make our technique of a superior umbilical fold incision impractical, as well as meaning that a “Yin-Yang” incision could not be hidden in a small umbilicus. One potential solution to this problem is to dissect the fascia around an umbilical incision and then place separate ports through the abdominal wall at different sites, thus facilitating the placement of the laparoscope and instruments without the need for a specialised insertion device [2, 5–7]. The technique described could very successfully be employed to facilitate this by exposing the linea alba superior to the umbilicus and dissecting a little more laterally.

Our study is clearly limited. We have employed this technique in only a small number of patients, and have subjectively assessed the cosmetic result, rather than seeking independent opinion to assess cosmetic outcome. In addition, our description of this technique is limited to those children who were of a sufficient size, with an appropriate umbilicus, to allow a 20 mm SILS port to be accommodated.

## 5. Conclusion

We have demonstrated the aesthetic benefits of utilising a superior umbilical fold incision for SILS in children.

## Figures and Tables

**Table 1:** Table showing the operative procedures performed over a one-year period using the technique described.

Procedure performed	No. of cases
Appendicectomy	10
Ovarian/paraovarian cystectomy	5
Palomo procedure	2
Nephrectomy	2
Heminephrectomy	1
Meckel's diverticulectomy	1
